# Managing childhood fever and pain – the comfort loop

**DOI:** 10.1186/1753-2000-1-7

**Published:** 2007-08-02

**Authors:** Jacqui Clinch, Stephen Dale

**Affiliations:** 1 Consultant paediatric rheumatologist and chronic pain specialist, Pain Management Unit, Southmead Hospital, Bristol, UK; 2 Analgesics, Reckitt-Benckiser, Nottingham, UK

## Abstract

Parents can transmit their anxiety to their child, and just as children can pick up on parental anxiety, they can also respond to a parent's ability to stay calm in stressful situations. Therefore, when treating children, it is important to address parental anxiety and to improve their understanding of their child's ailment. Parental understanding and management of both pain and fever – common occurrences in childhood – is of utmost importance, not just in terms of children's health and welfare, but also in terms of reducing the economic burden of unnecessary visits to paediatric emergency departments. Allaying parental anxiety reduces the child's anxiety and creates a positive feedback loop, which ultimately affects both the child and parent.

In this review, the integral role of parental perception of the child's condition and the efficacy of treatment in the management of childhood fever and pain will be discussed.

## Background

Parents inevitably worry about their children when they are ill. Increased parental anxiety has been demonstrated to result in increased anxiety of their children [1]. Heightened patient and parental anxiety increases the perception of pain and makes its treatment more difficult [2]. In surgery paediatric patients, parental preoperative anxiety is of particular importance for the anesthesiologist as increased parental anxiety results in increased anxiety in their children [3]. This heightened anxiety response, in turn, leads to immediate postoperative maladaptive behavioural responses in the children, such as nightmares, separation anxiety and eating disturbances [1,4,5]. One reason for the heightened anxiety in parents of children undergoing surgical procedures may be level of information received by parents regarding the anaesthesia and the surgical procedure. In painful procedures, such as surgery, one way to alleviate parental anxiety is to provide more information about the procedure, the anaesthesia and the post-operative period to the parents [6,7]. Interventions that may treat or prevent childhood preoperative anxiety, and hence decrease parental anxiety, and possibly also decrease the development of negative behaviours post surgery include sedative premedication, parental presence during anaesthetic induction, behavioural preparation programs, music therapy, and acupuncture [8].

Parents' anxiety related to their children's health is demonstrated in common childhood conditions such as fever [9] and pain [10] associated or independent of fever. Parent concerns about infant pain may contribute to parental stress [10]. Recurrent abdominal pain has been associated with symptoms of anxiety among children and their mothers [11]. Improved health education is required to allay parents fear and anxiety and promote a more appropriate fever management at home [9,12]. Parents also have unmet information needs about infant pain and wish greater involvement in their infant's pain care [10]. Parent concerns about infant pain also contribute to parental stress [10]. A study by Kai highlighted the primary concerns of parents when young children become acutely ill and has explored why they worry about them [13]. The study demonstrated that two factors appeared fundamental in shaping parents' responses: parents' sense of personal control when faced with an acute illness in their child and the perceived threat posed by an illness [13]. Better understanding of parents' concerns in acute illness may promote effective communication between health professionals and parents.

### Impact of parental anxiety on childhood pain

As they grow, children will experience numerous episodes of pain. Assessing pain is an integral component of pain management; however, this is often challenging in young children, who lack effective communication skills. Typically, in children under the age of 2 years, pain must be assessed by using physiological measures and inferred subjectively from the child's facial expressions, body movements and vocalisation.

Eccelston and colleagues have developed a multidimensional measurement tool addressing the impact of adolescent chronic pain [14]. This is a validated tool that assesses anxiety, depression, functional disability, physical ability, coping strategies and somatisation in adolescents who have suffered ongoing pain. Similar measures were applied to the parents and this strongly illustrated the impact of parental anxiety and perception of functional disability and the subsequent rehabilitation of their child. Thus, the more disabled they saw their child and the greater their anxiety then the slower the rehabilitation of the young person. Goubert and colleagues developed a Pain Catastrophising Scale (PCS-P) to study parental catastrophic thinking about their child's pain in parents of schoolchildren (N = 205) and in a sample of parents of children with chronic pain (N = 107) [15]. In the clinical sample, parents' catastrophic thinking about their child's pain had a significant contribution in explaining childhood illness-related parenting stress, parental depression and anxiety, and the child's disability and school attendance, beyond the child's pain intensity. Tsao and colleagues reported a study of responses to anxiety-related questionnaires and laboratory pain tasks in 211 non-clinical parent-child pairs (104 girls, mean age = 12.4 years; 178 mothers) [16]. They found that children's anxiety was related to laboratory pain-intensity ratings but not to pain tolerance using three pain tasks; cold, heat and pressure. When Tsao et al examined the influence of parents' anxiety sensitivity or fear of their own arousal symptoms (Anxiety Sensitivity Index [ASI]) on their children's laboratory pain-intensity ratings, they found that parents' anxiety sensitivity predicted their child's anxiety sensitivity, which in turn predicted their laboratory pain intensity – but, only in girls. Since more than 85% of the parents were mothers, the findings indicate that the mother-daughter anxiety/sensitivity link may indirectly influence girls' pain responses.

Another study assessed the validity of the Protect Scale of the Adult Responses to Children's Symptoms (ARCS) Questionnaire with regard to 67 mothers' responses to their children's abdominal pain [17]. The results demonstrated that mothers' protective responses to children's abdominal pain complaints at home predicted subsequent health service use for gastrointestinal symptoms. Similarly, Levy et al examined the relative contributions of psychological symptoms of the mother, psychological symptoms of the child, severity of child abdominal pain and family stress to consultation in an observational study of 275 mothers of 334 children [18]. The results revealed that both the child's self-report of perceived pain severity (p < 0.001) and maternal psychological symptoms (p = 0.006) predicted consultation. The authors concluded that the decision to take a child to the clinic for abdominal pain is best predicted by maternal psychological distress and the child's perceived pain severity [18]. Hence, by addressing the impact of pain on the parents we could positively influence the child's progress.

Many parents also have misconceptions of pain and analgesics and their use in children. Zisk et al [19] studied the relationship between children's and parents' (n = 110) sociodemographic and personality characteristics and parents' perceptions of their children's pain. More than 70% of parents feared side effects of analgesia, 43% thought analgesics were addictive, and 37% thought that the less often children receive analgesia, the better it worked. Less educated parents and parents of more sociable and more reactive children were more likely to indicate that they would avoid giving analgesia (Avoidance factor; p < 0.001). Parents with higher conscientiousness scores (NEO-FFI) and those with more impulsive children were more likely to perceive that analgesia was appropriate to use for child pain (Appropriate Use Attitude factor; p < 0.001).

One reason that may explain why parents find dealing with their child's pain so stressful is the obligation they feel to alleviate the suffering; therefore, it is their insecurity in dealing with the child's pain, rather than the pain itself that is more likely to generate such anxiety and stress in the parents [20]. In the day surgery setting, parental stress and anxiety has been shown to be associated with adverse post-surgery outcomes in their children. In one study, children with parents who found the day surgery to be stressful, experienced greater problems at home such as, fever, vomiting, sleep disorders, eating disorders and postoperative pain, compared with children who had calm parents [20]. In another study conducted in the day surgery setting, the children who were more upset at induction of anaesthesia were those who were accompanied by extremely anxious parents; more importantly, the level of preoperative parental anxiety was reflected in the children's behaviour and fears a week later [21]. In children with chronic pain, a holistic approach to pain management, which incorporates measures to address both child and parent anxiety, has proved to be beneficial [22]. Based on these observations, measures that address parental anxiety in the acute setting – thereby decreasing their uncertainty and concurrently increasing their sense of security and comfort – may also prove to be of benefit in the overall well-being of the child.

### Impact of parental anxiety on the management of childhood fever

Fever is a component of the febrile response stimulated by a complex series of physiological reactions in response to exogenous pyrogens such as infectious agents and toxins [23].

Most paediatricians define fever as a rectal temperature greater than 38.0°C or an oral temperature above 37.8°C [24]. However, whilst the measurement of temperature is commonly undertaken by healthcare professionals, elevated temperature alone should not represent a signal for pharmacological intervention. Anti-pyretic therapy should therefore be used to promote the comfort of children with associated symptoms such as pain and discomfort.

Fever and pain occur together frequently in childhood conditions. However, fever alone is one of the most common complaints of childhood, eliciting great concern and anxiety in parents. Up to 30% of visits to paediatric clinics or emergency departments are due to fever [24]. Parental anxiety about childhood fever is due partly to misconception – they believe that fever is a disease rather than a symptom or sign of illness [25]. Some parents believe that treating their child's fever could counteract illness [26]. Parents also fear the development of febrile convulsions, cerebral damage and even death in untreated children [27,28]. A recent paper reported a review which draws together findings from studies targeting parents' temperature-taking, antipyretic administration, attitudes, practices and information-seeking behaviours [29]. The results demonstrated that parental knowledge about normal body temperature and the temperature that indicates fever is poor. Mild fever is misclassified by many as high, and they actively reduce mild fever with incorrect doses of antipyretics. Although some parents acknowledge the benefits of mild fever, concerns about brain damage, febrile convulsions and death from mild to moderate fever persist irrespective of parental education or socioeconomic status [29]. Increased use of antipyretics to reduce fever and waking sleeping febrile children for antipyretics or sponging reflects heightened concern about harmful effects of fever. The overall conclusion of the authors of this paper was that over the course of 2 decades, little has changed in the knowledge, attitudes and practices of parents with regard to fever management. There is a need for interventions based on behaviour change theories to target the precursors of behaviour, namely knowledge, attitudes, normative influences and parents' perceptions of control [29].

Excessive concern about cerebral damage and other consequences of fever is also seen in healthcare professionals [25,30-32]. In one study, less experienced paediatricians were more likely to succumb to parental anxiety and subsequently give them inappropriate advice – in this case alternating ibuprofen and paracetamol – compared with more experienced paediatricians [33]. Similarly, lack of attention to evidence-based guidelines and misconceptions about fever have also been reported in less experienced nurses [31,34]. Key predictors of intentions to administer antipyretics to febrile children have been shown to be belief-based attitudes towards the agent and normative influences from anxious parents [32]. Another study demonstrated that up to 36% of nurses interviewed were not aware of the beneficial effects of fever [35]. They were also not aware that typically, children who have suffered from febrile convulsions have a benign prognosis [35].

Addressing parental anxiety is a key element in increasing their understanding of fever and how to manage it. A meta-analysis of several studies has demonstrated that parental knowledge about normal body temperature is poor, as is their knowledge of the temperature that indicates fever, with many parents misclassifying mild fever as high [29]. A substantial number of parents also wake their sleeping child to administer antipyretics, or check the temperature of the child too frequently – every hour or less [28,35]. Such behaviour can severely disrupt the rest and convalescence of ill children. Of greater concern is the inappropriate use of antipyretics. Many parents tend to administer the wrong dose of the agents and/or administer them at incorrect dosing intervals [28,29]. In one survey, 57% of parents treated children with incorrect doses of antipyretic drugs. In 11% of the children treated, the daily dose was at a level that could cause severe toxicity [36]. In contrast, a Swiss study revealed that fever was often undertreated, especially by nurses and even more so by parents [30]. Underdosing may lead to unnecessary, repeated clinic and/or emergency room visits [36]. Another practice reported in a number of studies is alternating ibuprofen and paracetamol [28,29]. This procedure is also practised by numerous healthcare professionals, including doctors [33] and nurses [34]. However, the weight of the evidence regarding any additional benefit compared with the respective monotherapies remains unclear. Published studies have indeed reported either additional antipyretic benefit compared with paracetamol or ibuprofen monotherapy [37] or conversely no additive effect [33] when employing an alternating regime. Importantly, alternating the two agents can be confusing, potentially leading to incorrect dosing of either product, or double dosing [33].

### Managing parental anxiety

Based on these observations, it is clear that parental knowledge of the treatment of fever must be improved. As there is no evidence that fever (not hyperthermia) causes any harm; therapy is usually aimed at promoting comfort rather than reducing temperature [38], hence, antipyretics with longer control of fever are likely to promote less anxiety in parents. Purssell undertook a systemic search to identify all studies comparing the antipyretic effects paracetamol and ibuprofen in children [38]. Statistical meta-analysis showed that by 6 hours after administration ibuprofen was clearly superior resulting in a mean temperature 0.58 degrees C lower than paracetamol. This study demonstrated that both drugs are effective antipyretics but the longer action of ibuprofen may make it preferable in some circumstances [38].

Erroneous beliefs and undue concern in healthcare professionals could contribute to anxiety in parents and serve to perpetuate what has been termed 'fever phobia' in parents [30,31]. Gehri *et al *investigated how 24 medical and nursing staff treated 114 feverish children under 5 years of age in a paediatric emergency department and compared the findings with their theoretical knowledge, evaluating how they might contribute to fever phobia in parents [30]. The results showed good consistency in theoretical knowledge, but an excessive fear about cerebral damage was also shown by doctors. This belief likely contributes to the transmission of fever phobia to parents [30]. Another study sought to examine 88 paediatric emergency nurses' knowledge base regarding fever in children since they commonly educate parents on fever management [31]. Fifty-seven percent of these nurses considered seizures the primary danger to a febrile child while 29% stated permanent brain injury or death could occur from a high fever. Therefore, fever phobia and inconsistent treatment approaches occur among experienced paediatric emergency registered nurses. These phobias and inconsistencies subsequently could be conveyed to parents. In order to assure accurate parental education, paediatric emergency departments should educate their medical team regarding the management of fever in children. Studies have also shown the positive impact on parental perception of having healthcare professionals with a better understanding of symptoms, diagnosis, management, and lifestyle implications of other diseases that may affect children e.g. Long QT syndrome, asthma, congenital heart surgery [39-41], which may, in turn, affect the child and his or her prognosis with the disease. Therefore, healthcare professionals themselves could benefit from further guidance on the management of childhood fever. Such education and guidance are essential as poor or inappropriate management may potentially lead to adverse consequences in children.

The sense of loss of control when faced with a febrile child contributes to parental anxiety [27]. Parents' control refers to the adequate control of the observed effects of an illness and protecting their child from potential harm [13]. This control is conditioned by their knowledge, beliefs and experiences, which subsequently influences their evaluation and management of their child's illness [13].

Similarly to childhood fever, pain in children is also a significant source of anxiety for the parent and adequate pain management helps reduce parental anxiety, which may reduce the child's' anxiety and aid recovery [42]. This may include educating parents and providing suitable analgesia for pain management. A study investigating parental management, of 100 parents, of their child's pain at home following day surgery demonstrated that parents managed their child's pain in the home if provided with information and suitable analgesia on discharge [43]. Watt-Watson *et al *also examined 71 parents' perceptions and concerns about their child's acute pain experience [44], using questionnaire that focused on the child's pain intensity, the behaviours that indicated the child was in pain, and the parents' preparation for and involvement in the child's pain experience. The majority of parents asked for more information about and greater participation in procedures that caused their child pain [44].

Consequently, an important aspect of treating children suffering from fever or pain should be to share information with parents, thus empowering them. This will allow parents to feel confident and secure when faced with future episodes of fever or pain in their children. Treating a child without the intervention of a healthcare professional enables parents to feel that they are coping with the situation [26].

Further, management of acute pain in children and anxiety in parents can also be achieved with analgesia. An important goal of analgesia is the safe and efficacious control of emotional distress as well as pain [45]. Goals of pain therapy should include minimisation – if not elimination – of painful or stressful stimuli, prevention of anticipatory pain, and rapid control of acute pain [46]. Similarly, fever can be managed with antipyretics, though the primary purpose for intervening when a child has a fever is to increase the child's comfort and therefore care needs to be individualised, based on current knowledge of the effectiveness and risks of interventions [47].

## Conclusion

Parental anxiety about their child's illness and treatment is multifactorial; however, it must be addressed as part of a comprehensive strategy when treating ill children. Parental anxiety has an impact on their clinical judgement, their understanding of the condition, compliance with their child's treatment and subsequent recovery [48]. In the day surgery setting, it has been shown that parental anxiety has an adverse effect on the child's recovery postsurgery [1]. Knowledgeable parents remain calm and confident – this may have a positive impact on the child, improving the overall management of fever and pain in the child. This will increase the child's comfort and reduce anxiety, not only in the child but also in the parents, thus engendering a positive feedback loop. Consequently, providing consistent information (from doctors, nurses and other professional healthcare workers) to the parents, reassuring them and allaying their fears can have a positive impact on the recovery of the child. If parents perceive a given therapeutic agent or strategy to be more efficacious than others, they will feel more confident, secure and better able to cope. As parental anxiety can be partly explained by the transmission of undue anxiety from healthcare professionals, nurses and doctors can also benefit from clear, evidence-based guidelines on the management of childhood fever and pain.

## Competing interests

Dr Dale was an employee of Reckitt-Benckiser at the time of writing of this review article. Reckitt Benckiser is a pharmaceutical company and manufacturer of aspirin and ibuprofen.

## Authors' contributions

JC was responsible for developing the "pain" section of the article whilst SD was responsible for developing the "fever" section
